# Current Status of the New Antiepileptic Drugs in Chronic Pain

**DOI:** 10.3389/fphar.2016.00276

**Published:** 2016-08-25

**Authors:** Harpreet S. Sidhu, Akshay Sadhotra

**Affiliations:** Pharmacology, Maharishi Markandeshwar UniversityAmbala, India

**Keywords:** antiepileptics drugs, neuropathic pain, migraine, mechanism of action, efficacy

## Abstract

Antiepileptic drugs (AEDs) are extensively used worldwide to treat a wide range of disorders other than epilepsy, such as neuropathic pain, migraine, and bipolar disorder. Due to this situation more than 20 new third-generation AEDs have been introduced in the market recently. The future design of new AEDs must also have potential to help in the non-epileptic disorders. The wide acceptance of second generation AEDs for the management of various non-epileptic disorders has caused the emergence of generics in the market. The wide use of approved AEDs outside epilepsy is based on both economic and scientific reasons. Bipolar disorders, migraine prophylaxis, fibromyalgia, and neuropathic pain represent the most attractive indication expansion opportunities for anticonvulsant developers, providing blockbuster revenues. Strong growth in non-epilepsy conditions will see Pfizer’s Lyrica become the market leading brand by 2018. In this review, we mainly focus on the current status of new AEDs in the treatment of chronic pain and migraine prophylaxis. AEDs have a strong analgesic potential and this is demonstrated by the wide use of carbamazepine in trigeminal neuralgia and sodium valproate in migraine prophylaxis. At present, data on the new AEDs for non-epileptic conditions are inconclusive. Not all AEDs are effective in the management of neuropathic pain and migraine. Only those AEDs whose mechanisms of action are match with pathophysiology of the disease, have potential to show efficacy in non-epileptic disorder. For this better understanding of the pathophysiology of the disease and mechanisms of action of new AEDs are essential requirement before initiating pre-clinical and clinical trials. Many new AEDs show good results in the animal model and open-label studies but fail to provide strong evidence at randomized, placebo-controlled trials. The final decision regarding the clinical efficacy of the particular AEDs in a specific non-epileptic disorder should be withdrawal from randomized placebo trials rather than open-label studies; otherwise this may lead to off-label uses of drug. The purpose of the present review is to relate the various mechanisms of action of new AEDs to pathophysiological mechanisms and clinical efficacy in neuropathic pain and migraine.

## Introduction

Neuropathic pain is chronic pain caused by injury to, or disease of, the central and peripheral nervous systems, occurring as a result of a cascade of neurobiological processes, which lead to hyperexcitability in conducting pathways of somatosensory neurons. Hyperexcitability is also hallmark of epileptic activity, and antiepileptic drugs (AEDs) remain the treatment of choice in the clinical management of neuropathic pain (McQuay et al., 1995). It is now recognized that hyperalgesia (exacerbated responses to painful stimuli) and allodynia (pain produced by otherwise non-painful stimuli) develop as a result of the pathological plasticity of sodium and calcium channels in several areas in the peripheral and spinal cord afferent pain pathways (Rogawski and Loscher, 2004a). In addition, other mechanisms involved in neuropathic pain include increased activity in glutamate receptors and changes in GABA-mediated inhibition, as well as alteration of calcium influx into cells (Rogawski and Loscher, 2004b). Many of the AEDs have been Food and Drug Administration (FDA)-approved for neuropathic pain but many others are commonly used in an off-label manner. Carbamazepine (CBZ), gabapentin (GBP), and pregabalin (PGB) are currently the only three AEDs approved by the U.S. FDA and European Medicines Agency (EMA) for the treatment of neuropathic pain. The most commonly studied neuropathic pain subtypes include diabetic neuropathic pain (DNP), postherpetic neuralgia (PHN), and HIV-related neuropathic pain. Collectively, these three conditions were estimated to affect over six million people across the seven major pharmaceutical markets (USA, UK, Japan, France, Germany, Spain, and Italy) in 2010 (Nightingale, 2012) However, the total affected population is considerably larger owing to the number of additional off-label uses, such as neuropathic lower back pain, cancer-related neuropathic pain, complex regional pain syndrome and postoperative neuropathic pain.

Fibromyalgia is defined as widespread pain for longer than 3 months with pain on palpation at 11 or more of 18 specified tender points (Wolfe et al., 1990) and is frequently associated with other symptoms such as poor sleep, fatigue and depression (Wolfe et al., 2014). More recently, a definition of fibromyalgia has been proposed based on symptom severity and the presence of widespread pain which does not require palpation of tender points for diagnosis (Wolfe et al., 2010). Moreover, patients with neuropathic pain and those with fibromyalgia experience similar sensory phenomena (Koroschetz et al., 2011) and peripheral nerve fiber changes seen in neuropathic pain also occur in fibromyalgia (Oaklander et al., 2013; Üçeyler, 2013). Fibromyalgia is a common condition, with a global mean prevalence of 2.7% (Range 0.4–9.3%) and a mean in the Americas of 3.1%, in Europe of 2.5% and in Asia of 1.7% (Queiroz, 2013). Fibromyalgia is more common in women, with a female to male ratio of 3:1.

Migraine is the most disabling of the neurological disorders worldwide (Murray et al., 2012) with a lifetime prevalence of 15–20% (Stovner et al., 2006). It is characterized by recurrent attacks of headache accompanied by autonomic symptoms such as photophobia, phonophobia, nausea and vomiting. Approximately 90% of migraineurs experience moderate or severe pain, three-quarters have a reduced ability to function during the headache attacks and one-third requires bed rest during their attacks (Lipton et al., 2007). Therefore, migraine induces severe disability in the majority of sufferers, causing a considerable burden to migraineurs, their families and society (Ferrari, 1998; Michel et al., 1997).

Evidence-based guidelines on the drug treatment of migraine have been developed and published by the European Federation of Neurological Societies (EFNS). These guidelines suggest that prophylactic therapy should be considered for patients with migraine when quality of life, business duties, or school attendance are restricted; when attack frequency exceeds one per month; in case of a lack of response to acute drug treatment; and when frequency, long, or uncomfortable auras occur (Evers et al., 2009).

## Literature Search

The literature search was done on Cochrane, Medline, Ovid, and PubMed. The review is based on recent publications on the activity of new generations AEDs in chronic pain conditions such as neuropathic pain and migraine. The papers published in the recent years were preferred, with the large majority of the included articles being published from the year 2000 onward. More than 95% conclusions were derived from randomized controlled trials (RCTs) and Cochrane Database of Systematic Reviews. Cochrane reviews are regularly updated as new evidence emerges and in response to feedback, and the Cochrane Database of Systematic Reviews should be consulted for the most recent version of the reviews. Open-label trials, case-reports, non-English language articles and articles of limited value, with out-of-date results or with poor methodology, were excluded.

## Review Of Literature In Neuropathic Pain

The first-line treatment options for the treatment of various neuropathic pain conditions are the AEDs: CBZ, GBP, and PGB (Goodyear-Smith and Halliwell, 2009). Sodium channel-blocking CBZ is effective in pain states by virtue of the same selective blocking properties of high-frequency action potential neuronal firing that account for its anti-seizure activity (Rogawski and Loscher, 2004a). The gabapentinoids (GBP and PGB) relieve neuropathic pain by selectively binding to the Ca^2+^ channels subunit α2δ-1 in the dorsal root ganglia (DRG) of the spinal cord. The α2δ-1 is the main molecular target, act as extracellular auxiliary subunit of voltage-gated calcium channels particularly the N- and L-types. The major function of α2δ-1 subunits is to direct trafficking of pore forming α1 subunits of Ca^2+^ channels from the endoplasmic reticulum to the plasma membrane (membrane trafficking). Neuropathic pain, due to injuries to peripheral/central nervous system, is associated with over-expression of the α2δ subunits of calcium channels (N-types) in the DRG neurons and the dorsal horn neurons of the spinal cord (predominantly C-fibers). The binding of gabapentinoids to over-expressing α2δ_1_ proteins may be responsible for the down regulation of N-type calcium channels in the spinal cord and pain attenuating effects in neuropathic pain (Taylor, 2009; Kukkar et al., 2013). The α2δ_1_ is also over-expressed within the area of the brain associated with nociceptive processing, such as dorsal raphe, periaqueductal gray, locus coeruleus (LC) and amygdala (Taylor and Garrido, 2008).

Gabapentinoids also have the ability to block anterograde trafficking of α2δ_1_ from DRG neurons to the pre-synaptic terminals of the dorsal horns resulting in reduced neurotransmitter release and spinal sensitization. Reduced Ca^2+^ entry at the presynaptic afferent terminal is expected to depress neurotransmission (Taylor, 2009) by decreasing the release of neurotransmitters such as glutamate, calcitonin gene related peptide (CGRP) and substance P that are involved in neuropathic pain progression (Kukkar et al., 2013).

The mechanism of action of gabapentinoids at the cellular level and after neuropathy has been the subject of much debate. Not a single molecular mechanism explain all aspects of analgesia, however, from the molecular and transgenic studies strongly support α2δ_1_ is the sole molecular target for the analgesic actions of gabapentinoids drugs.

Nerve injury induced up-regulation of α2δ_1_ in sensory neurons and spinal dorsal horn led to increased frequency, but not amplitude, of miniature excitatory post synaptic currents mediated mainly by AMPA/Kainate receptors at physiological membrane potentials, and also by NMDA receptors upon depolarization without changing the excitability of wide-dynamic-range neurons to high intensity stimulation in animal model. Together, these findings support a mechanism of α2δ_1_-mediated spinal sensitization in which elevated α2δ_1_ causes increased pre-synaptic glutamate release that leads to reduced excitation thresholds of post-synaptic dorsal horn neurons to innocuous stimuli. This spinal sensitization mechanism may be contributed to neuropathic pain states partially (Nguyen et al., 2009; Zhou and Luo, 2014, 2015). The α2δ_1_ knockout mice model was showed that α2δ_1_ expression appears to be a rate-limiting factor in transmitting abnormal peripheral activity to central neurons and is key in shaping the initiation of neuropathic pain, but the absence of α2δ_1_ fails to prevent chronicity and is not essential in the maintenance of a neuropathic state (Patel et al., 2013).

Peripheral nerve injury is also associated with up-regulation of expression of thrombospondin-4 (TSP4) in spinal cord and DRG of the spinal cord. At one end, peripheral nerve injury up-regulates α2δ_1_ in peripheral sensory neurons and its central terminals, while at other end it’s increased the synthesis and release of TSP4 in spinal cord and DRG of spinal cord. TSP4 activates its receptor α2δ-1 on sensory afferent terminals in dorsal spinal cord to promote excitatory synaptogenesis and central sensitization that contributes to neuropathic pain states (Eroglu et al., 2009; Park et al., 2016). The TSP4/α2δ_1_-dependent processes are important in mediating central sensitization and chronic pain states. Although TSP4 is up-regulated within days after peripheral nerve injury in both DRG and dorsal spinal cord (Kim et al., 2012; Pan et al., 2015), there is a significant delay in peak α2δ_1_ up-regulation in the dorsal horn (weeks; Park et al., 2016), primarily due to time required for initial translocation of elevated α2δ_1_ from DRG to pre-synaptic central terminals of sensory afferents in the dorsal horn (Bauer et al., 2009). The subsequent interaction of elevated TSP4 with excess pre-synaptic α2δ_1_ in the dorsal horn then promotes aberrant excitatory synaptogenesis and dorsal horn neuron sensitization to maintain chronic pain states. It is now clear that processes responsible for the initiation of neuropathic pain may differ from those responsible for its long-term maintenance. Microglia activation may be primarily associated with pain onset and astrocytes may contribute to the persistence of pain over periods of months and years (Grace et al., 2014).

Gabapentinoids may also act supra-spinally to treat neuropathic pain by stimulating descending inhibition to produce anti-hypersensitivity in peripheral nerve injury. Peripheral nerve injury induces differential changes in the plasticity of GABAergic neurons in the LC (increase in GABA release) and spinal dorsal horn (decrease in GABA release) and gabapentinoids are reported to selectively reduce pre-synaptic GABA release in the LC, not in the spinal dorsal horn. The gabapentinoids mediated reduction in GABAergic activity in the LC is associated with an increased noradrenaline release that in turn suppresses neurotransmission of pain in the spinal cord via activation of α_2_ adrenoreceptors (LC provides a descending inhibitory noradrenergic input to the dorsal horn; Tanabe et al., 2005). This effect would be expected to reduce excitability and to impede the transfer of nociceptive information. But this effect of gabapentinoids is seen only *in vivo*. Recently, a study has shown that activation of Brain-derived neurotrophic factor-tropomyosin receptor kinase B (BDNF-trkB) signaling is essential for gabapentinoids mediated activation of descending inhibitory pathway involving α_2_ receptors (Kukkar et al., 2013).

Gabapentinoids are transported into the neuronal cytoplasm via a neutral amino acid transporter (Cheng and Chiou, 2006) where they bind to α2δ_1_ (Field et al., 2006; Bauer et al., 2009). Interruption of the interaction of α2δ_1_ with pore-forming α-subunits of Ca^2+^ channels reduces the trafficking and the appearance of functional channels at the cell surface. The resulting decrease in channel availability would be expected depolarization-induced Ca^2+^ influx and this has been suggested to reduce neurotransmitter release (Cheng and Chiou, 2006; Field et al., 2006; Bauer et al., 2010). This assumption later found to be invalid for gabapentinoids mechanism of action in neuropathic pain. It was found that neurotransmitter release process is independent of depolarization via Ca^2+^ influx through calcium channels expression in plasma membrane of nerve terminals (Hoppa et al., 2012; Biggs et al., 2014). At the cell surface GBP does not disrupt the interaction between α2δ_1_ and α_1B_ subunits (Cassidy et al., 2014). GBP also fails to inhibit the internalization rate of α2δ_2_ but may be disrupt rab11-dependent recycling from endosomal compartments consequently reducing calcium currents through this mechanism (Tran-Van-Minh and Dolphin, 2010). It is speculate that at the spinal level, acute gabapentinoids treatment targets channel cycling pathways. In the dorsal horn of the spinal cord α2δ_1_ subunits of voltage gated calcium channels (VGCCs) mediate forward trafficking of channels from the endoplasmic reticulum, and this process can be facilitated by protein kinase C. The descending facilitations from the brainstem activating presynaptic ionotropic 5-HT_3_ receptors in the spinal dorsal horn results in membrane depolarization and may have consequences for VGCCs activity. Gabapentinoids inhibits rab11-dependent recycling of endosomes, while having no effect on the interaction between α2δ_1_ and VGCCs at the membrane. An inhibition of channel recycling results in reduced channel expression at the synaptic membrane and a decrease in transmitter release. Gabapentinoids do not affect single-channel kinetics of VGCCs and have only modest effects on neurotransmission (Dooley et al., 2007). This mechanism of action is independent of mechanism of gabapentinoids through TSP4. This acute effect would be observed *in vivo* not *in vitro* (Suzuki et al., 2005; Rahman et al., 2009).

The epidermal growth factor (EGF)-like domains of TSP4 directly binds to α2δ_1_ and mediates its synapse-inducing activity via this receptor. The α2δ_1_ act as a neuronal TSP receptor that is required for CNS synapse formation. This function of α2δ_1_ is likely to be independent of calcium channel function and numbers. Gabapentinoids are the potent inhibitors of TSP/astrocyte-induced excitatory synapse formation in both *vitro* and *vivo*. GBP binding to α2δ_1_ involves a region just upstream of the von willebrand factor A (VWF-A) domain in α2. Therefore, it is unlikely that TSP and GBP compete for the same binding site. GBP binding to α2δ_1_ restricts the confirmation of the VWF-A domain and keeps α2δ_1_ in its inactive confirmation. This perturbs the TSP-α2δ_1_ interaction and inhibits activation of the synaptogenic signaling complex (Eroglu et al., 2009). A critical threshold concentration of GBP in the cerebrospinal fluid is required to be effective in blocking synapse formation. TSP4-induced dorsal horn neuron sensitization and behavioral hypersensitivity is a slow process that requires 4 days to reach the peak effects, GBP at a clinically relevant concentration can block these effects within 1 h (Park et al., 2016).

The actions of gabapentinoids on DRG or dorsal horn neurons take at least 17 h to develop *in vitro* (Heblich et al., 2008; Hendrich et al., 2012; Biggs et al., 2014), which do not correlate with their rapid actions in animal model *in vivo*, where antiallodynic effects can be seen within 30 min of intraperitoneal injection (Patel et al., 2001; Field et al., 2006; Kumar et al., 2013). The rapid and slowly developing effects of gabapentinoids are well explained in a recent study. According to them, the α-subunits of VGCCs associate with α2δ_1_ subunits in the DRG and both are trafficked to the primary afferent nerve terminals in the dorsal horn neurons. The α2δ_1_ subunit is responsible for trafficking channels to the plasma membrane of nerve terminals. This result in up-regulation of functional calcium channels on plasma membrane and enabling their coupling with the neurotransmitter release machinery. The expressed channels are then removed from the membrane by endocytosis into endosomes, where they are targeted for recycling or degradation. In the control or uninjured nerve, where levels of α2δ_1_ are low, cycling of channels to and from the plasma membrane in nerve terminals are proceeds at a relatively slowly. However, this process takes place at a much more rapid rate, when α2δ_1_ is up-regulated either experimentally or as a result of nerve injury. Since gabapentinoids do not affect the rate of endocytosis, this renders surface expression of α2δ_1_ more liable and susceptible to inhibition by gabapentinoids. As a result we see the onset of actions within minutes rather than hours. In uninjured nerve, where α2δ_1_ is not up-regulated, gabapentinoids impediment of trafficking of α2δ_1_ –induced expression of calcium channels from cell bodies to nerve terminals. This result in gradual depletion of the surface expression of channels, as rate of endocytosis exceeds the rate of replenishment. These processes take about 17 h or more (Alles and Smith, 2016).

The gabapentinoids produce rapid effects in animal models, then why is it commonly reported that the drug effects take many days to appear in the clinic setting (Sharma et al., 2010; Parsons et al., 2015). One possibility is that in patients presenting with chronic neuropathic pain, α2δ_1_ is no longer up-regulated and other maladaptive process have taken over the maintenance of central sensitization. From the available literature, we did not get any information about the persistence of injury-induced α2δ_1_ up-regulation in either animal models or in patients. Since gabapentinoids are not universally effective, as many as 50% of treated patients do not experience pain relief with GBP (Moore et al., 2014). One of the reasons may be not all chronic pain conditions are associated with increased expression of α2δ_1_ and/or TSP4. It would be interesting to know whether α2δ_1_/TSP4 levels in individual patients would predict drug efficacy. So, further research in this field is urgently required to understand the protracted action of gabapentinoids in the clinic.

The starting dose of GBP is 900 mg/day given as three equally divided doses, increasing gradually up to a maximum of 3600 mg/day. The initial dose of PGB is 150 mg/day given as 2–3 divided doses. Based on patient response and tolerability, the dose may be gradually increased, to a maximum dose of 600 mg/day. GBP can also be formulated as an aqueous solution for injection, but this formulation is not licensed for treatment of any type of neuropathic pain or fibromyalgia. GBP has a half-life of 5–7 h. It is absorbed through a saturable transport system, so that absorption is not linear, and the transporter is found only in the proximal small bowel. This means that the drug needs to be administered at least three times daily to obtain plasma trough levels. Two new formulations have been introduced to improve oral bioavailability. The first is an extended-release gastro-retentive formulation, designed to provide continuous delivery at the optimal site of absorption over 8–10 h. The second formulation is extended-release prodrug (GBP enacarbil) that is absorbed through a high capacity transport system found throughout the intestine and then undergoes rapid hydrolysis to GBP. The human trials showed it to produce extended release of GBP with almost twice the overall bioavailability, especially when taken with a fatty meal. Due to the enhanced absorption of GBP enacarbil compared to GBP, the two drugs are not dose equivalent. PGB is related in structure to GBP. Compared to GBP, PGB is more potent, more quickly absorbed, and has greater bioavailability.

A recent Cochrane Library report (2014) reviewed 37 studies in 38 reports (5633 participants) that examined GBP (1200 mg/day or more) effect in 12 chronic neuropathic pain conditions (Moore et al., 2014) **(Table 1)**. In this report more than 84% of participants (13 studies) were in studies of PHN, painful diabetic neuropathy and mixed neuropathic pain. The other nine neuropathic pain conditions were studied in 922 participants, with the largest numbers in cancer-related neuropathic pain (356 participants), fibromyalgia (150 participants) and nerve injury pain (120 participants). Twenty-three studies had a parallel-group design and 14 had a cross-over design. Four studies used a gastroretentive, extended release formulations of GBP, and four others used an extended release prodrug, GBP enacarbil. Using the Initiative on Methods, Measurements, and Pain assessment in Clinical Trials (IMMPACT) definition of substantial benefit and moderate benefit model (Dworkin et al., 2008); data were analyzed.

**Table 1:** Efficacy outcomes with Gabapentin in PHN and DNP.

	Outcome	No. of studies	Participants	Improvement with GBP (%)	Improvement with placebo (%)	RR	NNT
lPHN	IMMPACT definition-any substantial pain benefit (at least 50% reduction from baseline)	7	2045	34	20	1.7 (1.4–2.0)	6.8 (5.4–9.3)
	IMMPACT definition-any at least moderate pain benefit (at least 30% reduction from baseline)	7	2045	44	27	1.6 (1.4–1.8)	5.7 (4.6–7.5)
lDNP	IMMPACT definition-any substantial pain benefit	6	1277	38	21	1.9 (1.5–2.3)	5.9 (4.6–8.3)
	IMMPACT definition-any at least moderate pain benefit	7	1439	52	37	1.4 (1.3–1.6)	6.6 (4.9–9.9)

*IMMPACT, initiative on methods, measurements, and pain assessment in clinical trials; PHN, postherpetic neuralgia; DNP, diabetic neuropathy; RR, relative risk; NNT, numbers-needed to treat.*

Adverse events occurred significantly more often with GBP. Persons taking GBP were experienced at least one adverse event (62%), withdraw because of an adverse event (11%), suffer somnolence (14%), dizziness (19%), peripheral edema (7%), and gait disturbance (9%). The author concluded that the amount of evidence for GBP in neuropathic pain conditions except PHN, DNP and mixed neuropathic pain is very limited, excluding any confidence that it works or does not work. The level of efficacy found for GBP is consistent with the efficacy estimates for other drug therapies in these conditions. There was no obvious difference between standard GBP formulations and recently introduced extended release or gastro-retentive formulations, or between different doses of GBP.

Pregabalin is marketed under different trademarks worldwide. US FDA approved the use of PGB for fibromyalgia in 2007, but the European Committee for Medicinal Products for Human Use (CHME) did not approve the use of PGB for fibromyalgia in Europe. The mode of action is similar to GBP, but has higher affinity for calcium channels is therefore used at lower doses. A recent Cochrane Library report (2008) reviewed 19 studies (7,003 participants) that examined PGB (300, 450, and 600 mg/day) effect in patients with PHN, DNP, central neuropathic pain and fibromyalgia (Moore et al., 2009). PGB was generally ineffective at 150 mg/day. Efficacy was demonstrated for dichotomous outcomes pooling together extent of pain relief, alongside with lower rates of lack of efficacy and discontinuations with higher dose. The best (lowest) number needed to treat (NNT) for each condition for at least 50% pain relief over baseline for 600 mg/day PGB (compared with placebo) was 3.9 (95% CI 3.1–5.1) for PHN, 5.0 (95% CI 4.0–9.9) for DNP, 5.6 (95% CI 3.5–14) for central neuropathic pain and 11.7 (95% CI 7.1–21) for fibromyalgia. With 600 mg PGB daily somnolence typically occurred in 15–25% and dizziness occurred in 25–46% of patients. Treatment was discontinued due to adverse events in 18–28%. The authors concluded that PGB provides a proven efficacy in neuropathic pain conditions and fibromyalgia, but with no benefit in acute pain scenarios. Due to the variability in patient’s response to PGB treatment, individualization is needed to maximize neuropathic pain relief and minimize adverse events.

Lacosamide (LCS) was approved in 2008 by both the USFDA and EMA as an adjunctive therapy for the treatment of partial-onset seizures with or without generalization in adults (17 years or older) patients with epilepsy, but later in 2014 it was additional approved by USFDA as monotherapy for the treatment of partial-onset seizures. It is not approved in the European Union as monotherapy. LCS selectively enhances slow inactivation of voltage-gated sodium channels (VGSC) without any effect on fast inactivation; whereas classical VGSC blockers such as phenytoin and CBZ produce fast inactivation. Slow inactivation is induced under conditions of sustained depolarization and repeated firing such as epilepsy or chronic neuropathic pain. LCS seems to enhance the slow inactivated state by altering the voltage-dependence of the VGSC subunit arrangement. LCS also modulates collapsing-response mediator protein-2 which may mediate neuronal plasticity. Dual mechanism of action might be an advantage in neuropathic pain. A recent Cochrane review on LCS efficacy in neuropathic pain and fibromyalgia included six studies: five (1,863 participants) in DNP and one (159 participants) in fibromyalgia (Hearn et al., 2012). All studies were placebo controlled and titrated to a target daily dose of 200, 400, or 600 mg. Study reporting quality was generally good, although the imputation method of last observation carried forward used in analyses of the primary outcomes is known to impart major bias, since adverse event withdrawal rates were high in the LCS studies. This coupled with small numbers of patients and events for most outcomes, meant that most results were of low quality, with moderate quality evidence available for some efficacy outcomes of LCS (400 mg/day).

In painful diabetic neuropathy, LCS (400 mg/day) provided statistically increased rates of achievement of “moderate” and “substantial” benefit (at least 30% and at least 50% reduction from baseline in patient-reported pain, respectively), and the patient global impression of change outcome of “much or very much improved.” For LCS 600 mg, there was no consistent benefit over placebo. There was no significant difference between any LCS dose and placebo for participants experiencing adverse effects, but adverse effect withdrawals showed a significant dose response. The number needed to harm (NNH) for adverse effect withdrawal was 11 at LCS 400 mg/day and 4 for 600 mg/day. The authors concluded that LCS has limited efficacy in treating diabetic neuropathy with limited beneficial effect at higher doses, but higher doses were associated with significantly more adverse event withdrawals. LCS has commonly off-label use in painful DPN. It is likely, therefore, that LCS is without any useful benefit in treating neuropathic pain; any positive interpretation of the evidence should be made with caution if at all. It is unknown if there will be further trials for neuropathic or other form of pain.

A recent Cochrane review showed non-convincing data for lamotrigine (LTG) in neuropathic pain. Twelve studies (1511 participants) were included, all with chronic painful neuropathic conditions (Wiffen et al., 2013b). None of the study provided first-tier evidence for an efficacy outcome. There was no convincing evidence that LTG is effective in treating neuropathic pain and fibromyalgia at doses of 200–400 mg daily. Almost 10% of participants of taking LTG reported a skin rash.

A recent Cochrane review investigated the efficacy of oxcarbazepine (OXC) in neuropathic pain. Four studies (779 participants) were included; three of them (634 participants) investigated the efficacy of OXC in painful diabetic neuropathy and one study (145 participants) in neuropathic pain due to radiculopathy (Zhou et al., 2013). Results for painful diabetic neuropathy showed that compared to the baseline the proportion of participants who reported a 50% reduction of pain scores after 16 weeks of treatment was significantly higher in the OXC group than the placebo group, with RR 1.91 (95% CI 1.08–3.39) and number of people needed to treat for an additional beneficial outcome (NNTB) 6.0 (95% CI 3.3–41.0). However, this positive outcome came from a single trial; other two negative trials did not provide data, which could not be included in meta-analysis. So the evidence from this review is incomplete and may not be widely acceptable. The authors concluded that more well designed, large, multicenter, randomized placebo, or active-controlled trials investigating OXC are needed.

A similar Cochrane review investigated the analgesic efficacy of topiramate (TPM) in neuropathic pain. Four studies (1684 participants) were included; three of them were in painful diabetic neuropathy (1643 participants) and one in radiculopathy (41 participants; Wiffen et al., 2013a). Doses of TPM were titrated up to 200 or 400 mg/day. None of the study provided evidence of efficacy in neuropathic pain whereas adverse event withdrawals were higher in active treatment group than placebo control.

Levetiracetam (LEV) is FDA-approved as add-on therapy in adult patients with focal seizures and myoclonic seizures. The drug assumed to act by modulating neurotransmitter release via binding to the vesicle protein SV2A (Lynch et al., 2004, 2008). However, its molecular mechanisms of action are still unknown. An analgesic effect of LEV in pain models might be explained by different pathophysiological mechanisms in various types of neuropathic pain as shown in different animals as well as in human neuropathic pain models (Enggaard et al., 2006; Archer et al., 2007; Ozcan et al., 2008; Sliva et al., 2008; Reda et al., 2016). However, the review of such results should also consider the increasing need for quality control and management in experimental research. Only few studies have tested LEV in chronic pain conditions. Some pilot and open-labeled case or cohort studies in humans showed LEV has analgesic effect in various neuropathic pain conditions (Hawker et al., 2003; Rowbotham et al., 2003; Price, 2004; Hamza et al., 2009). Recently, various studies demonstrated no analgesic effect of LEV in the randomized placebo-controlled and double-blind studies on painful polyneuropathy, post-mastectomy pain syndrome, central post-stroke pain, or spinal cord injury pain (Vilholm et al., 2008; Finnerup et al., 2009; Holbech et al., 2011; Jungehulsing et al., 2013). Further large randomized placebo-controlled trials are required to clarify the analgesic potential of LEV in neuropathic pain.

Zonisamide (ZNS) has a broad label use in Japan, while the regulatory bodies in the USA and Europe have approved it for use only as an adjunctive therapy for partial seizures in adults. ZNS has multiple mechanisms of action, one of them cause modulation of dopaminergic and serotonergic neurotransmission. Recently, animal model study investigated the role of serotonergic descending inhibitory pain pathways in antihyperalgesic effectiveness of ZNS in the streptozotocin-induced rat model for painful diabetic neuropathy (Bektas et al., 2014). The result of the study supports the role for ZNS in the management of DNP in all phases. Serotonin 5-HT_2A/2C_ and 5-HT_3_ receptors are involved in the antihyperalgesic effect of ZNS by enhancement of thermal threshold. A recent Cochrane review on ZNS included a single study treating 25 participants (13 ZNS, 12 placebo) with painful diabetic neuropathy over 12 weeks (Moore et al., 2015). The review found a lack of evidence suggesting that ZNS provides a pain relief in any neuropathic pain condition. This single available study was small and potentially subject to major bias. It cannot be regarded as reliable. Larger randomized, double blind, placebo-controlled trials on ZNS are required to find the true potential of this drug as analgesic.

Eslicarbazepine acetate (ESL) is a novel antiepileptic drug that is structurally related to CBZ and OXC but possesses a more favorable metabolic profile. Unlike CBZ, it is not susceptible to enzyme induction and unlike OXC (which is a prodrug of both the S- and the R-enantiomers of licarbazepine), ESL is a prodrug of predominantly the S-enantiomer of licarbazepine (McCormack and Robinson, 2009). Currently, there are no published clinical data on the analgesic properties of ESL; however, preclinical studies aimed at evaluating its efficacy in the treatment of migraine, postherpetic neuralgia and painful diabetic neuropathy have begun (Verrotti et al., 2014). A most recent animal study demonstrated that ESL produces dose-dependent antinociceptive effects in models of trigeminal, neuropathic and visceral pain in mice. ESL exhibited similar efficacy across all models but had a different potency in producing antinociceptive effects. It was the most potent in the writhing test and the least potent in the streptozotocin-induced diabetic neuropathy model. Study also demonstrated that antagonist of serotonergic receptors (5-HT_1B/1D_) and cannabinoid receptors (CB_1_/CB_2_) exerted dose-related inhibition of the antinociceptive effect of ESL, which implicates an involvement of these receptors in the antinociceptive action of ESL (Tomic et al., 2015).

Retigabine (RTG) or ezogabine is an antiepileptic drug, approved by the USFDA and EMA for the treatment of partial seizures in 2011. In 2013, the therapeutic use of RTG has been restricted by the EMA as an adjunctive treatment of drug-resistant partial onset seizures with or without generalization in adults. RTG has a unique mechanism of action that involves opening of neuronal Kv7.2–7.5 (formerly KCNQ 2–5) voltage activated K^+^ channels. These channels (primarily Kv 7.2/7.3) enable generation of the M-current, a sub-threshold K^+^ current that serves to stabilize the membrane potential and control neuronal hyperexcitability (Wuttke et al., 2005; Wang and Li, 2016). M-current channels are more negative than the action potential threshold. It means they are active at potentials below the threshold for action potential initiation and tend to increase rapidly in magnitude with depolarization. This creates a force opposing to depolarization, because the current does not inactivate but remains active during prolong periods of depolarization it tends to reduce firing frequency. Opening of these channels by RTG leads to hyperpolarization of the cell membrane, and a resultant decrease in cell excitability. A number of recent studies have reported that RTG can relieve pain-like behaviors such as hyperalgesia and allodynia in animal models of neuropathic pain (Takeda et al., 2011; Abd-Elsayed et al., 2015; Cai et al., 2015). Although several different types of neuropathic pain animal models have been developed and extensively studied, but still we fails to identify the common therapeutic molecular target among the voltage gated K^+^ channels (Kv7) in the neurons located in the nociceptive pathway. More, extensive research has been required to explore the exact role of Kv channel opener in the neuropathic pain.

Rufinamide was approved by the USFDA for adjunctive use in the treatment of seizures associated with Lennox-Gastaut syndrome in children 4 years or older and in adults. Exact mechanism of action is still unknown, but *in vitro* studies suggest that it act through modulation of VGSC. It appears to prolong the inactive state of VGSC by slowing the recovery of these channels from inactivation (Wier et al., 2011; Kharatmal et al., 2015). VGSCs are key determinants regulating action potential generation and propagation; thus, changes in sodium channel function can have profound effects on neuronal excitability and pain signaling. Pain sensations typically originate in sensory neurons of the peripheral nervous system which relay information to the central nervous system. VGSCs in sensory neurons play a crucial role in neuropathic pain. Electrophysiological and pharmacological studies have revealed that specific sodium channels subtypes particularly Nav1.3, Nav1.7, Nav1.8, and Nav1.9 are predominantly expressed and involved in the peripheral nociceptive neurons associated with pain signaling (Cummins et al., 2007; Theile and Cummins, 2011; Eijkelkamp et al., 2012). These subtypes of sodium channels are further classified into two main groups based on its sensitivity to tetrodotoxin (TTX). TTX-sensitive includes (Nav1.3, Nav1.7) and TTX-resistant includes (Nav1.8, Nav1.9; Elliott and Elliott, 1993; Rush et al., 1998). Rufinamide has a great affinity for TTX-resistant than TTX-sensitive channels (Kharatmal et al., 2015). Rufinamide attenuates allodynia response in animal models of inflammatory and diabetes induced neuropathic pain (Suter et al., 2013; Kharatmal et al., 2015). It has completed phase IV clinical trial, but results are not posted (ClinicalTrials.gov Identifier: NCT01212458, 2014). However, most of the sodium channel blockers that are currently available are often associated with cardio-toxicity and central nervous system side effects. Topical application, targeted drug delivery with minimize off-target side effects, development of isoforms specific VGSC blockers or development of sodium channel modulators that target specific patterns of sodium channel activity associated with problematic pain are the key approaches in the future research to tackle this problem.

## Review Of Literature In Migraine

The use of AEDs for the prophylactic treatment of migraine is theoretically warranted by several known modes of action, which relate either to the general modulation of pain system (Wiffen et al., 2010) or more specifically to systems involved in the pathophysiology of migraine. Migraine and epilepsy both are episodic disorders that share many clinical features and underlying pathophysiological mechanisms (Rogawski, 2012). The prevalence of migraine in populations of individuals with epilepsy is estimated at 8–24%, so that the risk of migraine is approximately twice that in the normal population. Similarly, the prevalence of epilepsy in individuals with migraine has been reported to be in the range of 1–17%, with a median of 5.9%, which is higher than the population prevalence of about 0.5–1% (Rogawski, 2012).

**FIGURE 1:**
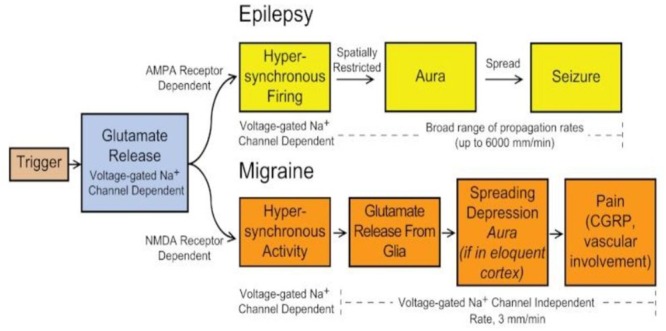
**Schematic illustration of the putative chain of cellular events in the evolution of an epileptic seizure and migraine attack, highlighting the similarities and differences.** In epilepsy, synaptic glutamate release acting through AMPA receptors is a trigger factor and synaptic activity is required (in most instances) for propagation. In migaine, synaptic glutamate acting through NMDA receptors is a trigger factor. Once established, synaptic activity may no longer be necessary and glutamate release from glia is the predominant factor that drives the advancing front of spreading depression. The spreading depression wave triggers the release of mediators that activate the trigeminovascular system, resulting in headache pain. Voltage-gated Na+ channel dependence (tetrodotoxin-sensitivity) implies the involvement of synaptic mechanisms. CGRP, calcitonin gene-related peptide (Adapted from Rogawski, 2012).

The pathophysiology of migraine involves abnormal neuronal activity such as hyperexcitability on the cortical surface during the aura phase and in the trigeminocervical complex (TCC) during the pain phase (Akerman et al., 2011). A causal association between migraine aura and headache is supported by evidence that both are linked to the phenomenon known as cortical spreading depression (CSD) of Leao (Chiossi et al., 2014). CSD is an expanding neural depolarization wave of cortical neurons, is the basis for the aura in migraine and the trigger for the subsequent headache pain. However, the initial event proceeding CSD is neuronal hyperexcitability associated with localized epileptiform discharges. CSD which occur spontaneously in the human cortex before the onset of headache; its pathogenesis is exactly not known. The susceptibility of its occurrence likely depends on genetic factors that render the cerebral cortex hyperexcitable thro channelopathy or abnormal excitatory/inhibitory imbalance (Chiossi et al., 2014). Glutamate is a critical mediator of the hyperexcitability in both focal seizures and migraine. In focal epilepsy, seizure generation and spread is mediated by synaptically released glutamate acting on AMPA receptors, whereas in migraine, triggering of CSD depends on NMDA receptors and spread does not require synaptic transmission. (**Figure 1**) Some AEDs prevent the occurrence of migraine attacks, while others not. AEDs act through a range of different mechanisms of action, which ultimately modulate neural systems involved in the pathophysiology of migraine. Therefore, it is not possible to dissect which mechanisms may be responsible for the therapeutic effect.

**FIGURE 2:**
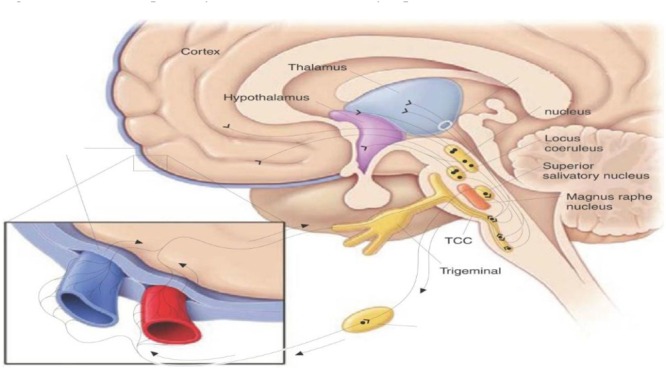
**Brainstem pathways that modulate sensory input.** The key pathway for pain in migraine is the trigeminovascular input from the meningeal vessels, which passes through the trigeminal ganglion and synapses on second-order neuron in the trigeminocervical complex (TCC). These neurons in turn project in the quintothalamic tract and, after decussating in the brainstem, synapse on neuron in the thalamus. Important modulation of the trigeminovascular nociceptive input comes from the dorsal raphe nucleus, locus coeruleus, and nucleus raphe magnus (Adapted from Goadsby and Raskin, 2015).

Cortical spreading depression is the key event that causes activation of the trigeminovascular system (TVS; Noseda and Burstein, 2013). The TVS pain pathway has both peripheral and central projections (Goadsby and Raskin, 2015) (**Figure 2**). The central projection involves TCC [spinal trigeminal nucleus + upper cervical spinal cord (C1–C2)] and VPM (ventral posteromedial) nucleus of thalamus. Third-order neurons lie in the VPM nucleus. Axons from these neurons project to the trigeminal area of the primary and secondary somatosensory (S1/S2) cortices, as well as the insula, suggesting a role in sensory-discriminative components of migraine such as location, intensity, and quality of pain. TCC also send projections to the posterior, lateral posterior/dorsal thalamic nuclei, which are in turn project to multiple cortical areas such as motor, parietal association, retrosplenial, somatosensory, auditory, visual and olfactory cortices, suggesting a role in motor clumsiness, difficulty focusing, transient amnesia, allodynia, photophobia, phonophobia, and osmophobia.

In the peripheral projection, spinal trigeminal nucleus (SpVC) plays a central role in the TVS of pain pathway. SpVC received input from higher centers. Fibers from SpVC project to superior salivatory nucleus, which in turn send fibers to sphenopalatine ganglion (SPG). Fibers originates from SPG innervates the meningeal blood vessels. These terminal nerve fibers release the chemical mediators such as neurokinin A, substance P and CGRP which is responsible for inflammation and vasodilatation of the meningeal and dural blood vessels. Clinically it correlates with throbbing headache. Such nociceptive information is transmitted via C and Aδ nerve fibers to first order neurons in the trigeminal ganglia (TG). Axons from TG project to SpVC. Second order neurons are located in the SpVC. Axons originate from second order neurons finally project to thalamic nuclei (Noseda and Burstein, 2013).

A large number of endogenous inflammatory mediators believed to be released during migraine are capable of activating and sensitizing peripheral and central trigeminovascular neurons. Peripheral sensitization mediates the throbbing perception of the headache (Strassman et al., 1996) whereas central sensitizations of second order neurons in the SpVC responsible for cephalic allodynia (Burstein et al., 2000, 2010). Overall, the data imply that migraine headache depends both on activation of the TVS by pain signals that originate in peripheral intracranial nociceptors, and on dysfunction of CNS structures involved in the modulation of neuronal excitability and pain.

There is extensive evidence from RCTs that divalproex sodium (valproate) and TPM are effective in preventing migraine attacks and both drugs are approved by the US FDA for this indication (Rogawski, 2008; Linde et al., 2013c,d).

The mechanism of action of GBP is to enhance GABA release through a non-vesicular mechanism. The pharmacological target of GBP is α2δ subunit of L-type voltage-gated calcium channel which modulate peripheral and central trigeminovascular dural nociceptive neurons, but not those in the thalamus (Hoffmann et al., 2014). The results from a recent meta-analysis suggest that GBP is not effective for the preventive treatment of migraine (Linde et al., 2013b). Total six trials were included, enabling comparison to placebo as well as dose comparisons of GBP or GBP enacarbil. All six trials had parallel-group designs and a median duration of the treatment phase of 12 weeks. The doses of GBP investigated in the trials were 900–2400 mg. No trial investigated PGB. Only one trial investigated GBP enacarbil. This drug is not approved by the FDA for the treatment of epilepsy but rather for the treatment of moderate-to-severe restless legs syndrome and postherpetic neuralgia in adults. The pooled results did not demonstrate a significant difference between GBP and either placebo or different GBP doses on the outcomes “headache frequency” or “responder rate.” The sole trial of GBP enacarbil failed to demonstrate a significant difference in responder rate across all dose ranges.

The main pharmacological actions of LTG are thought to be by inhibiting glutamate release, believed to be via blocking voltage-sensitive sodium channels. LTG is not effective for the preventive treatment of migraine (Linde et al., 2013a). The animal models studies showed that LTG was able to significantly decrease the number of repetitive CSDs in rats (Bogdanov et al., 2011). As we discussed earlier that CSD is clinically correlates with occurrences of aura symptoms of migraine. This data provide strong support that LTG is effectively relieve aura symptoms of migraine without relief in headache phase. However, this hypothesis was investigated in a small pilot study as well as an open clinical trial, suggested that LTG had efficacy in the reduction of migraine aura (Lampl et al., 1999, 2005). Further clinical trials are needed to confirm the efficacy of LTG for aura treatment.

A single parallel-group trial examined ZNS vs. TPM (200 and 100 mg, respectively) and found no significant difference between them in reduction of headache frequency from baseline during the third month of treatment (Linde et al., 2013a). This should be investigated further in placebo-controlled trials because conducted trial had incomplete outcome data. The absence of a significant difference in effect between ZNS and TPM is not proof of an actual effect of ZNS.

Levetiracetam has been reported to be effective in two open-label studies (Brighina et al., 2006; Pakalnis et al., 2007). The controlled trial reported in chronic daily headache, in which some patients clearly had chronic migraine, was negative (Beran and Spira, 2011). In another placebo-controlled study, showed that LEV at a dose of 1000 mg/day was superior to placebo in relieving migraine frequency, severity and functional disability (*p* < 0.0001; Verma et al., 2013). A recent randomized, double-blind, parallel-groups, placebo-controlled study explored the efficacy of LEV (500 mg/day) versus sodium valproate (500 mg/day) versus placebo in the prevention of migraine. The results showed that there was no statistically significant difference between LEV and valproate groups in the mean change of migraine frequency. However, it was significant when comparing LEV or valproate to placebo (*p* = 0.02 for both; Sadeghian and Motiei-Langroudi, 2015). Both placebo-controlled trials have methodological issues (small sample size), high risk of reporting and attrition bias. A meta-analysis based on the existing data suggests that LEV is not effective in the preventive treatment of migraine (Linde et al., 2013a). Large-scale randomized studies are needed to verify these findings. Large future trials should be encouraged on line with recommendations of the International Headache Society with regard to both trial design and reporting of data.

A randomized, double-blind, placebo-controlled trial was conducted by UCB which is a multinational biopharmaceutical company to evaluate the efficacy of LCS (100 and 300 mg/day) compared to placebo in reducing the frequency of migraine. The primary efficacy variable was the mean reduction of migraine rates during the 14-week maintenance period compared to the average frequency during the 4-week baseline period. The trial did not meet its primary endpoint. However, a reduction in headache frequency was consistently observed in all treatment groups (ClinicalTrials.gov Identifier: NCT00440518, 2014).

A recent randomized, double-blind, parallel-group, placebo-controlled, multicentre trial explored the efficacy, safety and tolerability of OXC (1200 mg/day) versus placebo in the prevention of migraine (Silberstein et al., 2008). There was no statistically significant difference between the OXC and placebo groups in the mean change of migraine frequency, indicating that OXC is not effective in the prevention of migraine.

With regard to other AEDs, no data from controlled trials are available for ESL, rufinamide, RTG, and perampanel for the preventive treatment of migraine.

Glutamate is the primary excitatory neurotransmitter throughout the peripheral and central nervous systems, and receptors mediating its actions represent candidate targets that have been explored to varying degrees in preclinical and clinical studies. Glutamate acts on both G protein-coupled metabotropic receptors (mGluRs) as well as three families of ligand-gated ionotropic receptors (iGluRs), the *N*-methyl-D-aspartate (NMDA), α-amino-3-hydroxy-5-methyl-4-isoxazolepropionic acid (AMPA), and kainite receptors (KARs). These receptors are found in areas of the central and peripheral nervous system that are important for the transmission of pain. During a migraine attack, levels of glutamate increase and activate these receptors, facilitating the transmission of pain impulses.

Tezampanel (LY-293558), an AMPA and kainate receptor antagonist, and was studied in a phase II trial. The trial was sponsor by Torreypines Therapeutics Company. The trial design was a double-blind, placebo-controlled, parallel-group multicenter study to assess the safety, tolerance and efficacy of a single subcutaneous dose of tezampanel (40, 70, or 100 mg) in patients with acute migraine. The primary endpoint was percentage of patients in each treatment group who experience a decrease in pain from moderate or severe intensity pre-dose (baseline) to mild or no pain 2 h after study drug administration and prior to use of rescue medication. There was statistically significance at the dose of 40 mg versus placebo (*p* = 0.03). At other two doses, there was no statistically significance on primary endpoint (ClinicalTrials.gov Identifier: NCT00567086, 2007).

BGG492 (selurampanel) is an AMPA receptor antagonist. This molecule was investigated in randomized, double-blind, proof-of-concept study to assess the efficacy of BGG492 (250 mg) versus placebo and sumatriptan (100 mg) in patients with acute migraine pain. However, Proof-of-concept criterion was not met (≥ 25% BGG492 subjects with a primary response vs. placebo at two time points). BGG492 was comparable to sumatriptan in terms of pain-free response (Gomez-Mancilla et al., 2014).

## Discussion

Anticonvulsants drugs represent the most investigated drugs among all other treatment options for the treatment of chronic pain. But still it is unclear, under which pharmacological mechanism of action responsible for their therapeutic effects in chronic pain. It is an important research agenda why some AEDs have good analgesic efficacy, while others have analgesic failure status. For example Valproate, TPM, LTG, ZNS, and CBZ all are sodium channel antagonists but only valproate and TPM have analgesic efficacy in the preventive treatment of migraine. Similarly, among all AEDs, GBP and PGB specifically target the α2δ type 1 subunit of calcium channels and both of them seem to have analgesic properties, which are confirmed in several randomized, placebo-controlled trials. On the other hand, many other AEDs have confirmed analgesic effect, but have no effect on calcium channels. Therefore, understanding the exact pathophysiology of disease for which AEDs may work is the most important step before initiating AEDs. It is also important to know the reasons, why some patients respond to a particular drug, while others do not. It suggests that future preclinical studies should focus on the detailed mechanisms and sites of action that define the clinical efficacy of AEDs. Certainly, in the future, dissecting out further how the anticonvulsants that do not work differ mechanistically from those that do will almost certainly provide more specific and novel approaches toward the development of new treatments in this regard.

Many new third generation AEDs have been marketed during recent years: rufinamide, ESL, RTG, LCS and perampanel. In all cases, AEDs are initially approved by the US FDA and the EMA for epilepsy and then subsequently for non-epilepsy disorders. For example, this happened for CBZ, LTG, and VPA in bipolar disorder or, more recently, for PGB in fibromyalgia in the USA and generalized anxiety disorder in Europe.

Anticonvulsants have a long history of off-label use; CBZ and valproate were widely used, for example, as alternative and adjunctive treatments to lithium for mood disorders, before they received regulatory approval. The increased off-label use of AEDs other than FDA-approved conditions is potentially dangerous. The data supporting the use of AEDs for such conditions is entirely based on case reports, small open-label series, or controlled studies that are limited by sample size and statistical power or by methods biased toward positive findings. For example, prescribing TPM for the treatment of major depression or anxiety is misleading. Data solidly show that TPM helps patients lose weight, but no data demonstrate a beneficial effect of drug in affective disorders. We think case reports and open-label trials are valuable for directing further research, they are generally not sufficient as the basis of treatment decisions. We give classical example of GBP (Neurontin) off-label uses: bipolar disorder, diabetic neuropathy, restless leg syndrome, trigeminal neuralgia, migraine, attention deficit disorder, low back pain, aggressive behavior associated with dementia, hot flushes associated with tamoxifen therapy and treatment of drug and alcohol addiction. At present more than 80% of clinical cases, this drug use as off-label. The drug approved by the US FDA in 1993 as an adjunctive therapy in the treatment of partial seizures with and without secondary generalization in patients over 12 years of age with epilepsy. The FDA subsequently approved it in 2000 as adjunctive therapy for the treatment of partial seizures in pediatric patients 3–12 years of age, as well as for the management of postherpetic neuralgia in adults (2004). In Europe, GBP is currently approved for similar indications. Because of wide spread off-label use of anticonvulsants, in 2008 US FDA has issued a warning on anticonvulsants that these drugs may increased the risk of suicide and suicidal thoughts.

Reasons behind off-label uses:

(1) Deceptive and illegal marketing practices: The pharmaceutical companies adopt strategies which strongly influence the prescribers such as funding dinners, conferences, and medical education seminars where presentations were made on off-label uses. Other methods include like company’s sales representatives encouraging doctors for promoting their products for off-label uses and paying doctors honoraria for use of their names on ghost written scientific articles. The classical example of this Dr. David Franklin, a former employee of Warner-Lambert, has filed a lawsuit in the district court of Massachusetts against the Multinational Pharmaceutical Company Pfizer for unethical promotion of Neurontin (GBP) for off-label uses for which there was not good evidence. Later the company pled guilty to the charge of illegally marketing the drug. Pfizer also admitted that it had used an illegal marketing strategy to promote Neurontin for off-label uses. For this illegal practice the company was fined $240 million.
(2) Unawareness among clinical practitioners: Most of the practitioners who are not in touch with teaching or scientific research, being unaware of on- and off-label indications.
(3) Trial design: Evidence from clinical practice and experience indicates that a few patients can achieve good results with AEDs, despite the RCT design fails to demonstrate greater efficacy than placebo for those AEDs. In such circumstance, practitioner may look for off-label use when there are limited treatment options available. At this stage, we need new trial design which can detect low but significant response rate. Enriched enrolment randomized withdrawal (EERW) designs carry a significant importance particularly in CNS drugs. Such designs can detect long-term efficacy and safety of the drug. They may be helpful whether intervention works or not. The classical example of this is FREEDOM trial of PGB in fibromyalgia. The efficacy of PGB in fibromyalgia was established through EERW trial.
(4) Off-label use of drug should be reserved for patients who have failed standard treatment options and drug established some degree of evidence in the RCTs. For example, GBP use in diabetic neuropathy and migraine prophylaxis.

## Conclusion

Different types of pathophysiological processes are involved in different types of chronic pain conditions, but one of the common process in all of them is neuronal hyperexcitability. The main targets for the action of the AEDs include enhancement of GABAergic inhibition, decreased glutamatergic excitation, modulation of voltage-gated sodium and calcium channels and effects on intracellular signaling pathways. All of these mechanisms are of importance in controlling neuronal excitability in different ways, such as in neuropathic pain where voltage-gated ion channels are important, in migraine abnormal excitatory/inhibitory imbalance and in bipolar disorder where intracellular signaling pathways are important. Many AEDs show a wide spectrum of activity and seem to affect these processes selectively.

None of the mechanisms of AEDs seem to be preferable to the others, as all of them decrease hyperexcitability, for example; valproate are effective in migraine and bipolar disorder, but it is not approved for the treatment of neuropathic pain. A better understanding of the mechanism of action of AEDs in different disorders will be provided by increased knowledge of the underlying molecular deficits in each disorder, and clinical investigations that prove efficacy of the AEDs in various disorders.

Future challenges include evaluating the third generation AEDs in chronic pain conditions, and designing trials for children and adolescents with migraine and neuropathic pain. The activation of the TSP4/α2δ_1_-dependent processes is required for TSP4-induced central sensitization that leads to pain state development. Blocking this pathway may be a novel strategy for development of target-specific analgesics for chronic pain management. The focus of future research should be on differentiating between the main mechanisms of action of the different drugs, and in considering the drugs of choice for optimal treatment of the various pain disorders.

## Author Contributions

We both contributed equally to the design, literature of search, and writing of the review.

## Conflict of Interest Statement

The authors declare that the research was conducted in the absence of any commercial or financial relationships that could be construed as a potential conflict of interest.
